# A Non-atherosclerotic Heart Tears Apart: A Case of Spontaneous Coronary Artery Dissection in a Healthy Postmenopausal Woman

**DOI:** 10.7759/cureus.25459

**Published:** 2022-05-29

**Authors:** Vishal Phogat, Subash Nepal, Hani Kozman

**Affiliations:** 1 Internal Medicine, Upstate University Hospital, Syracuse, USA; 2 Cardiology, Upstate University Hospital, Syracuse, USA

**Keywords:** spontaneous coronary dissection, cardiac death, sudden cardiac, post partum acs, nste-acs, scad types, scad management

## Abstract

Due to limited understanding and knowledge of spontaneous coronary artery dissection (SCAD), it is typically believed to affect young peripartum women. We present a case of a post-menopausal older woman who suffered an acute non-ST segment elevation myocardial infarction (NSTEMI), secondary to a SCAD of the right posterior descending artery (PDA), after strenuous exercise. As the patient was hemodynamically stable and without signs of ongoing ischemia, she was managed conservatively. SCAD should be in differentials for patients presenting with acute coronary syndrome (ACS) who have no or few cardiovascular atherosclerotic risk factors. SCAD can be missed due to low suspicion of ACS in young and healthy patients. ACS from SCAD is often misdiagnosed and/or mismanaged as atherosclerotic ACS. Increasing awareness about this condition can lead to earlier diagnosis and prevention of sudden cardiac deaths. As most cases of SCAD can be managed conservatively, differentiating it from atherosclerotic ACS can reduce unnecessary reperfusion procedures and complications thereof.

## Introduction

SCAD is a non-traumatic separation of the coronary arterial wall, which is not iatrogenic and not associated with atherosclerosis. It is an underdiagnosed and rare cause of acute coronary syndrome (ACS) and sudden cardiac death. Due to limited data and understanding of SCAD, it is often misdiagnosed and managed as atherosclerotic ACS [1]. We present a case of a 60-year-old female who presented with ACS after exertion at the gym. She was found to have a SCAD of the right posterior descending artery (PDA) on coronary angiography.

## Case presentation

A 60-year-old Caucasian female with a past medical history of breast cancer (in remission), and generalized anxiety disorder, presented to the emergency department (ED) with a one-day history of a sudden onset of chest tightness and intermittent episodes of palpitations. 

She first experienced some chest discomfort and palpitations earlier in the morning when she was working out at the gym. She described it as a constant burning sensation in her chest up to her throat and a pounding of the heart. These symptoms did not improve upon resting. She then went home and rested for a couple of hours. The chest tightness persisted on waking, and she continued to experience intermittent episodes of palpitations. Her Fitbit showed her heart rate to be 120-130. She also experienced diaphoresis, tremulousness, and lightheadedness, which prompted her to present to the ED for evaluation. The patient mentioned that she usually experiences intermittent and brief episodes of palpitations, but this episode was longer and more persistent. She had a Holter monitoring 20 years ago for palpitations and chest discomfort, which did not show any significant arrhythmias requiring intervention. The patient had no other prior cardiac history of myocardial infarction (MI), congestive heart failure, valvular heart disease, hypertension, diabetes, or hyperlipidemia. She had a 1.5-pack-year history of cigarette smoking, and she quit smoking 31 years ago. She did not report any recreational drug use. Multiple family members had breast cancer, but no cardiac problems were reported.

**Figure 1:**
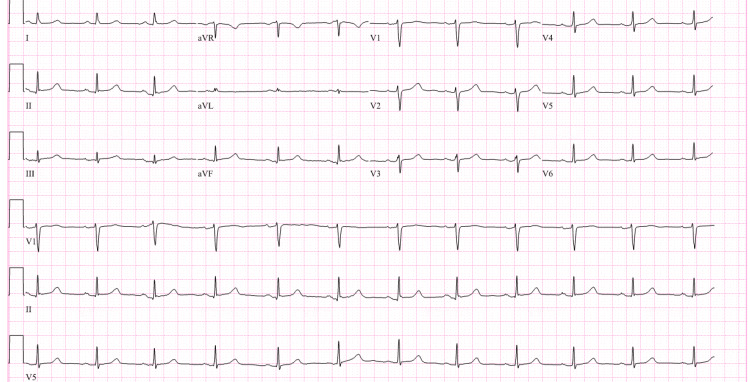
Electrocardiogram showing a normal sinus rhythm Normal sinus rhythm with a ventricular rate of 66 beats per minute was seen on an electrocardiogram. No acute ST segment or T wave changes were noticed.

In the ED, the patient was hemodynamically stable, and the physical exam was unremarkable. However, the patient was orthostatic upon standing up and received intravenous (IV) fluids. High-sensitivity troponin was elevated at 442 ng/L (normal <14 ng/L). Other labs, including electrolytes, D-dimer, and lipid panel, were normal. Hemoglobin A1c was also normal at 5.4%. Chest X-ray showed no acute pathology. Electrocardiogram (EKG) showed a normal sinus rhythm (Figure 1). 

On telemetry, the patient was noticed to have a brief run of 16 beats of non-sustained ventricular tachycardia (NSVT) during an episode of palpitations. The patient was started on aspirin, high-intensity statin, and an IV heparin drip for management of ACS. Beta-blockers were not started due to orthostatic hypotension. High-sensitivity troponins peaked up to 1179 ng/L, followed by a downtrend in the subsequent hours. A transthoracic echocardiogram showed hypokinesis of the inferior and inferoseptal walls (Videos 1-2). 

**Video 1:** Echocardiogram showing hypokinetic mid and distal inferior wall An echocardiogram clip shows the apical two-chamber view with the hypokinetic mid and distal inferior wall.

**Video 2:** Echocardiogram showing hypokinetic mid inferoseptal and distal inferior wall An echocardiogram clip shows the apical four-chamber view with hypokinetic mid inferoseptal and distal inferior wall.

**Figure 2:**
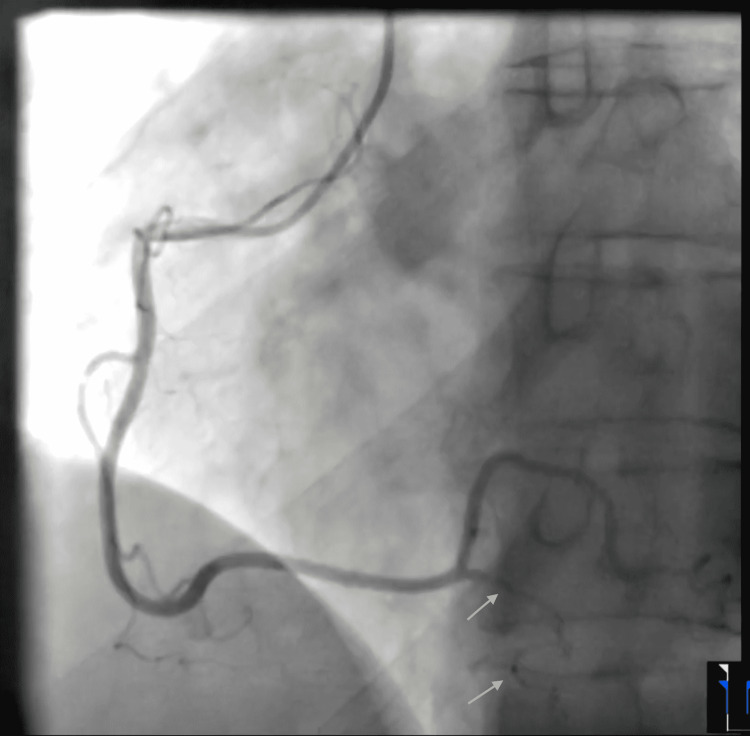
Coronary angiogram LAO view showing a right posterior descending artery spontaneous coronary artery dissection Right coronary angiogram LAO view shows smooth and diffuse stenosis and string-like appearance of the right posterior descending artery (pointed with two arrows), suggestive of type 2 spontaneous coronary artery dissection. LAO - left anterior oblique

The patient's chest pain resolved after a few hours. Coronary angiography showed a diffuse long and smooth stenosis of the right posterior descending artery (PDA), consistent with a type 2 SCAD and otherwise normal angiography (Figure 2, Videos 3-8).

**Video 3:** Selective right coronary angiogram, left anterior oblique view, showing a spontaneous coronary artery dissection involving the posterior descending artery A selective angiogram of the right coronaries in the left anterior oblique (LAO) view shows a diffuse narrowing of the right posterior descending artery, consistent with a type 2 spontaneous coronary artery dissection.

**Video 4:** Selective right coronary angiogram, right anterior oblique view, showing a spontaneous coronary artery dissection involving the posterior descending artery A selective angiogram of the right-sided coronaries in the right anterior oblique (RAO) view shows string-like diffuse stenosis of the right posterior descending artery, suggestive of type 2 spontaneous coronary artery dissection.

**Video 5:** Selective left coronary angiogram, right anterior oblique caudal view, shows normal left-sided coronaries A selective angiogram of the left-sided coronaries in the right anterior oblique caudal view shows angiographically normal left anterior descending artery, left circumflex artery, obtuse marginal artery, and the diagonals.

**Video 6:** Selective left coronary angiogram, left anterior oblique cranial view, showing normal left-sided coronaries A selective angiogram of the left-sided coronaries in the left anterior oblique cranial view shows angiographically normal left main coronary artery, proximal left anterior descending artery, and the proximal left circumflex artery.

**Video 7:** Selective left coronary angiogram, left anterior oblique caudal view, showing normal left-sided coronaries A selective angiogram of the left-sided coronaries in the left anterior oblique caudal view shows angiographically normal left main coronary artery, proximal left anterior descending artery, and the proximal left circumflex artery.

**Video 8:** Selective left coronary angiogram, right anterior oblique cranial view, showing normal left-sided coronaries A selective angiogram of the left-sided coronaries in the right anterior oblique view shows angiographically normal left anterior descending artery and the diagonals.

Further imaging (like intravascular ultrasonography or optical coherence tomography) or percutaneous coronary intervention (PCI) was not deemed necessary. The patient was discharged home the following day with aspirin, clopidogrel, beta-blocker, and a close follow-up with outpatient cardiology.

## Discussion

SCAD was first reported in 1931 at an autopsy of a woman aged 42 who died suddenly after experiencing chest pain. SCAD has an unknown true prevalence. It is often missed due to a low suspicion of ACS in young women and a lack of clinician familiarity with SCAD. Some recent case series suggest that it may be associated with up to 1-4% of all ACS cases and is the most common cause of pregnancy-associated MI [2-4]. The mean age was described to be 51.8 years for women with SCAD, and it is increasingly being reported in postmenopausal and older women [5]. Most patients with SCAD do not have conventional risk factors for coronary artery disease (CAD). A predisposing arterial disease, such as fibromuscular dysplasia (FMD), is associated with most cases [6,7]. Postpartum status, multiparity, connective tissue diseases, systemic inflammatory conditions, and hormonal therapy have been identified as potential predisposing factors [7]. Cardio-circulatory stressors like intense Valsalva-type activities, labor and delivery, intense exercise or emotional stress, recreational drugs, and aggressive hormonal therapy can provoke acute SCAD, particularly with an underlying predisposing arteriopathy [6]. 

The pathophysiology of SCAD is not fully understood, but two theories have been proposed. First, intimal tear as the primary event generating a false lumen and an intramural hematoma (IMH) [8]. Second, spontaneous hemorrhage and IMH from vasa vasorum leading to an intimal rupture into the true lumen [9]. IMH within the tunica media can compress the true lumen resulting in MI. A periadventitial inflammatory reaction (with a predominance of eosinophils) has been described on histology, which may help distinguish SCAD from iatrogenic postmortem dissection. The majority of patients diagnosed with SCAD experience symptoms and signs of ACS, chest pain being the most common presenting complaint. From 2% to 19% of patients with ST-elevation MI (STEMI) related to SCAD can suffer cardiogenic shock [2]. Ventricular arrhythmias and sudden cardiac death can also be the presenting feature in some patients. 

SCAD should be suspected in young patients, women, and patients with no or few cardiovascular risk factors who present with ACS. Coronary angiography is the first-line diagnostic imaging method for diagnosing SCAD. The most commonly affected coronary vessel is the left anterior descending artery (LAD), followed by the branches of the circumflex artery, followed by the branches of the right coronary artery (PDA and posterolateral arteries) [6]. SCAD can be classified into three different types based on coronary angiography. Type 1 refers to arterial wall staining with multiple lumens. Type 2 is most common and refers to a long diffuse and smooth narrowing on angiography due to an IMH. Type 3 mimics atherosclerosis with focal or tubular stenosis. Adjuvant diagnostic strategies like intravascular ultrasonography (IVUS) and optical coherence tomography (OCT) can be a helpful aid to angiography. However, they have potential risks (like iatrogenic coronary dissection, an extension of an existing dissection, and occlusion of the true lumen) and thus, should only pursue when the diagnosis is uncertain from angiography alone (such as type 3 lesions), and the vessel diameter is large enough for intra-coronary imaging [1]. Cardiac computed tomography (CT) angiography can help identify proximal lesions but can miss a substantial number of cases of SCAD involving distal coronary arteries or the side branches [10].

Due to the limited clinical experience and rarity of the disease, there are no guidelines on optimal management of SCAD, and the treatment recommendations are based on expert consensus. Conservative management is preferred in most cases as spontaneous time-dependent healing was observed in the majority of the cases (70-97%) managed conservatively [1, 11]. This includes the use of long-term aspirin, a short course of clopidogrel, and statins in patients with dyslipidemia [12]. Beta-blockers have a long-term benefit and reduce the recurrence of SCAD [13]. Percutaneous coronary intervention (PCI) can be challenging in SCAD. The high complication and failure rates have been reported with revascularization, including a high likelihood of in-stent restenosis and late stent mal-apposition with a temporal resolution of IMH. PCI or coronary artery bypass grafting (CABG) should be considered in patients who present with acute MI (along with the symptoms of ongoing ischemia or hemodynamic compromise) or a dissection involving the left main artery [1]. Cardiac rehabilitation is encouraged after discharge. 

Our patient was a healthy postmenopausal older female who presented with an acute non-ST segment elevation MI and was found to have a smooth and diffuse narrowing of the right PDA on coronary angiography, which was consistent with a type 2 SCAD. No further imaging (IVUS or OCT) was deemed necessary. She had no prior cardiac history, history of trauma, or atherosclerotic risk factors. Her cholesterol levels, including the low-density lipoprotein (LDL), were normal. Coronary angiography also did not show any atherosclerotic disease. She mentioned strenuous exercise at the gym earlier in the morning when she first experienced chest tightness, which could have precipitated the SCAD. Our patient was hemodynamically stable, without any signs of ongoing ischemia. Therefore, she was managed conservatively. Statins were not started as the patient did not have CAD or dyslipidemia. She was advised to closely follow up with outpatient cardiology, where they could consider further imaging and possible genetic studies to rule out fibromuscular dysplasia and connective tissue disorders. This case highlights the increasing incidence of SCAD in older and postmenopausal women, as compared to the original belief that SCAD primarily affects young peripartum women.

## Conclusions

The prevalence of SCAD has been increasing in older patients, including postmenopausal women. SCAD should be considered in the differential diagnosis for a patient presenting with ACS with no or few cardiovascular atherosclerotic risk factors. Increasing awareness about SCAD can reduce the overall morbidity and mortality from the complications of the reperfusion procedures for ACS secondary to SCAD since most of these cases can be managed conservatively. Further case reports, case series, and clinical trials are needed to better understand the etiology, epidemiology, and pathophysiology of SCAD, which can also help in establishing treatment guidelines.
